# Tic Douloureux of the Face and Head

**Published:** 1844-06

**Authors:** 


*Tic Douloureux of the Face and Head.*—M. Ducros recently communicated to the Academy the details of some well-marked cases of this distressing disease, which were rapidly cured by the use of strong ammonia, applied to the palate, gums, &c., with a camel-hair brush, so as to occasion a profuse discharge of tears and saliva. He requested the physicians of several of the metropolitan hospitals to repeat his experiments on a large scale; and the results of their trials have been, he says, most satisfactory.

The strong aqua or liquor ammoniæ, taken internally, will be found to be a most valuable remedy in many cases of neuralgic suffering about the face and head, odontalgia, severe nervous headache, &c. The best mode of administering it is to mix from 20 to 40 drops in a cupful of very thick gruel, and to take this at bed time, or whenever the paroxysm of pain is present. The ammonia must be well blended with the gruel, else it will irritate very painfully the inside of the mouth and throat. It should produce a profuse salivation and lachrymation. In very severe or obstinate cases, it may be applied outwardly at the same time.—*Med. Chi. Rev.*, Jan.

